# Inflamma-miR-21 Negatively Regulates Myogenesis during Ageing

**DOI:** 10.3390/antiox9040345

**Published:** 2020-04-23

**Authors:** Maria Borja-Gonzalez, Jose C. Casas-Martinez, Brian McDonagh, Katarzyna Goljanek-Whysall

**Affiliations:** 1 School of Medicine, Physiology, National University of Ireland, H91 W5P7 Galway, Ireland; 2 Institute of Ageing and Chronic Disease, University of Liverpool, Liverpool L7 8TJ, UK; 3 The Medical Research Council Versus Arthritis Centre for Integrated Research into Musculoskeletal Ageing (CIMA), Liverpool L7 8TJ, UK

**Keywords:** microRNA, muscle, aging, sarcopenia, cachexia, regeneration, miR-21, IL6, IL6R

## Abstract

Ageing is associated with disrupted redox signalling and increased circulating inflammatory cytokines. Skeletal muscle homeostasis depends on the balance between muscle hypertrophy, atrophy and regeneration, however during ageing this balance is disrupted. The molecular pathways underlying the age-related decline in muscle regenerative potential remain elusive. microRNAs are conserved robust gene expression regulators in all tissues including skeletal muscle. Here, we studied satellite cells from adult and old mice to demonstrate that inhibition of miR-21 in satellite cells from old mice improves myogenesis. We determined that increased levels of proinflammatory cytokines, TNFα and IL6, as well as H_2_O_2_, increased miR-21 expression in primary myoblasts, which in turn resulted in their decreased viability and myogenic potential. Inhibition of miR-21 function rescued the decreased size of myotubes following TNFα or IL6 treatment. Moreover, we demonstrated that miR-21 could inhibit myogenesis in vitro via regulating IL6R, PTEN and FOXO3 signalling. In summary, upregulation of miR-21 in satellite cells and muscle during ageing may occur in response to elevated levels of TNFα and IL6, within satellite cells or myofibrillar environment contributing to skeletal muscle ageing and potentially a disease-related decline in potential for muscle regeneration.

## 1. Introduction

Progressive muscle atrophy during ageing (sarcopenia), or as a result of conditions such as cancer (cachexia) results in frailty, a decline in strength and a decrease in the quality of life of older people. The mechanisms underlying muscle wasting are complex and largely associated with underlying pathological process [1]. Several common mechanisms have been proposed to be associated with a loss of muscle mass and function during both ageing and cachexia, such as increased levels of proinflammatory cytokines and chronically elevated levels of reactive oxygen species (ROS) [2]. Chronic redox stress and inflammation have been proposed to contribute to muscle atrophy through regulating satellite cell function, and therefore muscle regeneration during ageing and cachexia [3,4,5,6,7]. Regeneration of adult skeletal muscle is largely dependent on satellite cell viability and functionality [8]. Ageing- and cachexia-related changes in satellite cell number and properties, such as susceptibility to apoptosis and ability to proliferate, resulting in impaired regenerative capacity have been shown in humans and rodents [9,10,11,12,13,14].

Among molecular mechanisms suggested as downstream mediators of elevated levels of ROS and pro-inflammatory cytokines are changes in gene, as well as gene regulatory molecules, such as the expression of microRNAs [3,15,16,17]. microRNAs (miRNAs, miRs) are short, non-coding RNAs, which regulate gene expression at the post-transcriptional level. microRNAs provide high-throughput mechanisms for controling cellular mRNA and protein content through regulating the expression of multiple genes in response to changes in the intra- and extracellular environment. miRs have been shown to regulate many biological processes, including skeletal muscle development, regeneration and ageing [15]. microRNAs guide RISC (RNA-induced silencing complex) to partially complementary sequences, usually within the 3′UTR of target mRNAs. miR binding to its target(s) results in degradation of the mRNA and/or translational block resulting in a decrease in protein levels. Muscle-specific miRs, also referred to as “myomiRs”, are important regulators of skeletal muscle function [18,19,20,21,22,23,24]. Changes in microRNA expression in muscle have been demonstrated during ageing and disease [12,24,25,26,27,28,29]. Satellite cell-specific knock-out of Dicer, an enzyme responsible for generation of the majority of mature miRs, in a mouse model resulted in mild myofiber atrophy [30].

miR-21 has been proposed a circulating marker of inflammation during ageing: inflamma-miR [31,32,33,34] and is also classified as an oncomiR due to its role in cancer progression [35]. The role of miR-21 has been reported in cachexia, where it is synthesised and exported from tumour cells and transported to skeletal muscle via exosomes resulting in muscle atrophy [28]. Moreover, miR-21 is also expressed in skeletal muscle and has been shown to contribute to muscle atrophy following denervation, regulating YY1 and EIF4E3 proteins [36]. Furthermore, miR-21 has been identified as playing an important role in muscle fibrosis during Duchenne muscular dystrophy [36,37]. miR-21 has also been shown to regulate skeletal muscle development in pigs via regulating PI3K/AKT/mTOR pathway [38].

This study investigated the involvement of miR-21 in the regulation of ageing-related decline in muscle regeneration and therefore potentially contributing to age-related loss of muscle mass and function. miR-21 is upregulated in muscle and satellite cells during ageing and its expression is elevated in the presence of ROS and pro-inflammatory cytokines: IL6 and TNFα. Moreover, changes in the expression of miR-21 during muscle regeneration are disrupted in regenerating muscle of old mice. We have demonstrated that miR-21 is a negative regulator of myogenic differentiation of satellite cells in vitro through regulation of viability and myogenic potential. Interestingly, inhibition of miR-21 using a specific antimiR in primary myoblasts in the presence of TNFα or IL6, rescued the decreased viability and myogenic potential of myoblast phenotype. Finally, we demonstrate that miR-21 regulates the expression of IL6R in primary myoblasts, as well as the levels of PTEN. miR-21 upregulation also results in localisation of FOXO3 in the nucleus and upregulation of Gadd45, suggesting miR-21 may regulate proapoptotic pathways. We hypothesise that the elevation of IL6 and TNFα during ageing and potentially cachexia results in increased expression of miR-21 and contributes to defective muscle regeneration resulting in muscle atrophy. 

## 2. Materials and Methods

### 2.1. Mice

The study was performed using muscle from male wild type C57Bl/6 mice (adult: 6 months old; old—24 months old). Mice were obtained from Charles River (Margate). All mice were maintained under specific-pathogen free conditions and fed ad libitum a standard chow and maintained on a 12-h light–dark cycle. For muscle regeneration, mice under isoflurane anaesthesia were injected with barium chloride (1.2% *w*/*v* in saline) into the right tibialis anterior. Mice were culled by cervical dislocation and tissues dissected immediately, frozen and stored at −80 °C. Ethical approval was received from the University of Liverpool Animal Welfare and Ethical Review Body (AWERB, PE80AB60F). Experiments were performed in accordance with UK Home Office guidelines under the UK Animals (Scientific Procedures) Act 1986. For each experiment, *n* = 3–6 biological replicates were used.

### 2.2. Satellite Cells Isolation

Satellite cells from adult and old mice used in this manuscript were obtained during a previous project [12]. Satellite cells were isolated using FACS (Flourescence-activated cell sorting) sorting as previously described [12]. Briefly, skeletal muscle isolated from the hind limbs of two male mice per sorting was treated with 1.5 U/mL collagenase D, 2.4 U/mL dispase II and 2.5 mM CaCl_2._ Satellite cells were sorted as α-7 Integrin^+^, Sca1^−^, CD45^−^ and CD31^−^. Doublets and hematopoietic and endothelial cells (CD45^+^ and CD31^+^) were excluded from the sorting gates. A pure population of satellite cells negative for Sca1 and highly positive for α-7 integrin was isolated (CD45^−^, CD31^−^, Sca1^−^ and α 7 Integrin^+^).

### 2.3. Satellite Cell Transfection and Myogenesis 

miR-21 function in satellite cells was studied in satellite cells located on isolated single myofibers. Satellite cells become activated, proliferate, migrate out of the fibres and differentiate [12]. Single fibres from mice EDL muscle were isolated using collagenase I (400 U/mL) at 37 °C, rocking. Isolated fibres were investigated under the microscope to discard broken fibres. Fibres were next plated in matrigel-covered wells in 12-well dishes. The cultures were maintained in DMEM media (Appendix A Table A1) supplemented with 20% foetal bovine serum, 10% horse serum and 1% penicillin/streptomycin and transfected with miR-21 mimic/antagomiR at 100 nM concentration 1 and 3 days post plating to enhance transfection efficiency. The formation and quantification of new myotubes was assessed 10 days following myofiber isolation via MF20 immunostaining [12].

### 2.4. Isolation of Primary Myoblasts from Mouse Skeletal Muscle

Primary myoblasts from adult (6 months old) and old (24 months old) mice were prepared from EDL muscles following single fibre isolation as previously described [12,39]. Briefly, EDL muscle from both legs was digested with 1.5 U/mL collagenase D, 2.4 U/mL dispase II and 2.5 mM CaCl_2_. Digested muscle was filtered and spun to remove undigested tissue and plated on surfaces covered with 10 µg/mL laminin and incubated with DMEM media with 20% FBS, 10% horse serum, 1% l-glutamine and 1% penicillin/streptomycin at 37 °C and 5% CO_2_. Primary myoblasts were grown in DMEM media supplemented with 10% FBS, 1% l-glutamine and 1% penicillin/streptomycin [39].

### 2.5. Cell Culture of Primary Myoblasts

Primary myoblasts were cultured as described previously [39]. Myogenic differentiation was induced by placing 90% confluent cells in DMEM supplemented with 2% horse serum and 1% penicillin/streptomycin (differentiation media; DM). Myoblast differentiation was examined after 5 days by immunostaining for myosin heavy chain: MF20 antibody concentrate was used at 1:100 dilution [12]. Briefly, cell were fixed in ice-cold methanol for 5 min, blocked in 10% horse serum for 1 h, incubated with primary (MF20) antibody, washed 3× PBS, incubated for 1 h in anti-mouse-488/532 antibody, washed 3× PBS and mounted onto cover slips. Differentiating cells were treated with either IL6 (0.2 ng/mL), TNFα (25 ng/mL) or H_2_O_2_ (50 µM) during the time course of differentiation. To study proliferation and viability, myoblasts were switched to DM once 50% confluent. IL6 (0.2 ng/mL), TNFα (50 ng/mL) or H_2_O_2_ (50 µM) treatment was performed for 3 days, subsequently an MTT assay, which measures metabolic activity, was performed. Images were analysed using ImageJ (Appendix A Table A2). Morphological analysis, measurement of myotube area was assessed as described [20].

### 2.6. Transfections of Primary Myoblasts

Myoblasts were transfected with 100 nM miRNA-21 or antimiR-21 (Qiagen) using Lipofectamine 2000^TM^ [20]. Mock-transfected cells served as controls unless otherwise stated. Transfection efficiency was 40–70% as per qPCR analyses, depending on the molecule transfected (Figure S1a, [20]). 

### 2.7. Real-Time PCR and Western Blotting

RNA isolation and quantitative real time RT-qPCR were performed using standard methods. RNA was isolated using Trizol as per manufacturer’s protocol. cDNA synthesis (mRNA) was performed using 500 ng RNA and SuperScript II (Appendix A Table A3). cDNA synthesis (microRNA) was performed using 100 ng RNA and miRscript RT kit II as per manufacturer’s protocol [12]. qPCR reaction was set up using miRScript SybrGreen Mastermix or Qiagen Quantitech SybrGreen Mastermix in a 20 µL reaction. Expression relative to β-2 microglobulin, 18S and 26S (geometric mean; mRNA) or Rnu-6 and/or Snord-61 (microRNA) was calculated using the delta delta Ct method.

Protein lysis and Western blots were done as described [24]. Primer sequences are listed in Table 1. Homogenised protein lysates were diluted in Laemmli buffer. 20 µg of protein was loaded and proteins were separated on 12% SDS PAGE gels. Proteins were next transferred using a semi-dry blotter and stained with Ponceau-S to visualise equivalent loading. Membranes were next blocked in 5% milk in TBS-T for 1 h at room temperature. Next, membranes were washed in TBS-T and incubated with primary antibodies at a dilution of 1 in 1000 in blocking buffer (please see Table 2 for antibody details). Li-Cor Biosciences anti-rabbit/mouse secondary antibodies (Li-Cor Biosciences) were diluted 1 in 10,000 in TBS-T and visualised using Li-Cor Biosciences Odyssey Fc. Each membrane was used twice—after initial detection of antigen, the membranes were stripped in stripping buffer (glycine, SDS, Tween 20 pH 2.2.) for 30 min, washed in TBST, blocked and reprobed for a different antigen as described above.

### 2.8. MTT Assay and Live/Dead Staining of Mouse Myoblasts

Myoblast metabolism was assessed by MTT assay to determine the number of live cells [12]. Briefly, cells were transfected as indicated, cultured in low serum media (DMEM supplemented with 2% horse serum and 1% Pen/Strep) above and cell viability as per metabolic activity measured was assessed 72 h later. Myoblast death was investigated by live/dead staining [12]. Live cells were washed in PBS and stained with acridine orange/ethidium bromide: PBS (1:1000). Within an hour of the staining images were taken.

### 2.9. Figure Preparation and Statistics

Images were assembled from raw images using Adobe Photoshop CC2017. To maintain the eventual differences and allow the images to be comparable with each other, brightness or contrast, if adjusted, changes were applied to all images of the panel. Western blot images were quantified using Image J (version 1.51, NIH, USA: https://imagej.nih.gov/ij/). Graphs were created in GraphPad Prism 8 software (GraphPad Software, San Diego, USA). Pair-wise comparisons were performed using a Student *t*-test, multiple comparisons were performed using one-way ANOVA.

## 3. Results

### 3.1. miR-21 Expression Is Upregulated during Ageing

**Figure 1:**
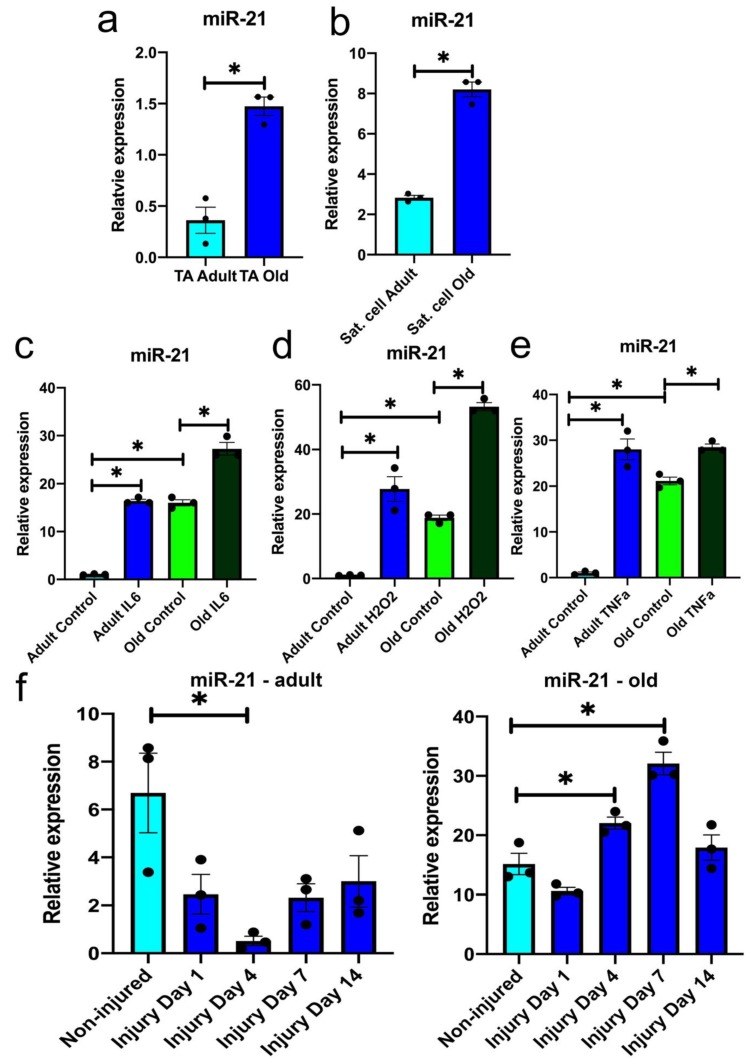
miR-21 expression is regulated in muscle during ageing and regeneration. (**a**,**b**) qPCR demonstrating changes in miR-21 expression in tibialis anterior (TA) muscle (**a**) and satellite cells (**b**) during ageing. (**c**–**e**) qPCR demonstrating upregulation in miR-21 expression following the treatment of primary myoblast from adult and old mice with 0.2 ng/mL interleukin 6 (IL6; **c**), 50 µM H_2_O_2_ (**d**) or 25 ng/mL TNFα, respectively, for 72 h. (**f**,**g**) qPCR demonstrating changes in miR-21 expression following mouse TA injury with barium chloride in the adult (**f**) and old (**g**) mice; day 0—non-injured muscle; day 1, 7, 14 and 21 days post-injury. • indicates individual replicates. Error bars show SEM, * *p* < 0.05 (compared to adult or non-injured control, respectively); *n* = 3 biological replicates. Expression relative to Rnu-6 (miR-21) is shown.

Circulating miR-21 (inflamma-miR-21) levels have been previously demonstrated to be elevated during ageing and correlate with inflammation [31,32]. However, the role of miR-21 in muscle and specifically muscle regeneration, in the context of age-related chronic inflammation is not known. To investigate whether miR-21 may play a role in defective muscle regeneration during ageing, we examined miR-21 expression in the whole muscle (tibialis anterior; TA) and FACS-sorted satellite cells from adult (6 months old) and old (24 months old) mice. miR-21 expression was upregulated in both TA and satellite cells during ageing (Figure 1a,b). We next examined changes in miR-21 expression during muscle regeneration: following barium chloride injury of the TA muscle from adult and old mice. miR-21 was significantly downregulated at day 4 during regeneration of TA from adult mice (Figure 1f). However, following the injury of muscle from old mice, miR-21 was upregulated at days 4 and 7 post-injury suggesting a defective response of miR-21 to muscle injury during ageing (Figure 1g).

The increased expression of miR-21 in muscle from old muscle, both quiescent and following injury, may be due to age-related increase in pro-inflammatory cytokines, such as TNFα or IL6, within the muscle itself or within its local or systemic niche, or exposure to H_2_O_2_. The levels of cytokines and ROS are transiently upregulated following muscle injury and have been proposed to be chronically dysregulated during ageing and contribute to defective muscle regeneration [3,7,40,41]. Primary mouse myoblasts from adult and old mice were treated with H_2_O_2_, IL6 or TNFα for 72 h, respectively, and miR-21 expression was evaluated by qPCR. All three treatments resulted in elevated miR-21 levels in primary myoblasts from adult and old mice, however this upregulation was more prominent in myoblasts from adult mice as compared to control cells (Figure 1c–e). Based on these data, we hypothesised that miR-21 may be upregulated as a result of the effects of H_2_O_2_ and pro-inflammatory cytokines during ageing and muscle regeneration.

### 3.2. miR-21 Negatively Regulates Myogenic Potential of Satellite Cells

**Figure 2:**
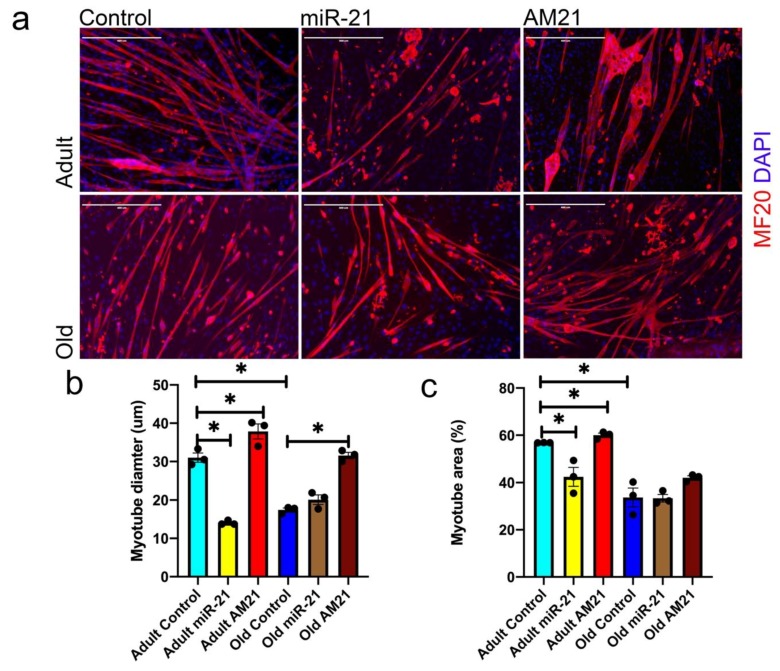
miR-21 negatively regulates myogenic differentiation of satellite cells in vitro. (**a**) Satellite cells migrating out of isolated single myofibers were transfected with miR-21 mimic (miR-21), anti-miR (AM21) or mock-transfected with Lipofectamine 2000 (control: mock-transfected cells); new myotube formation was established by myosin heavy chain immunostaining: MF20—red; DAPI—blue. Quantification of myotube diameter (**b**) and quantification of total myotube area (**c**; as % of the field of view) is shown. Error bars show SEM, • indicates individual replicates *, *p* < 0.05 (*—compared to adult/old Ctrl, respectively), *n* = 3. Scale bar = 400 µm.

Effective regeneration of the muscle depends on satellite cell viability, proliferation and differentiation. To establish the role of miR-21 in a physiologically relevant context, we investigated the potential of satellite cells migrating out of single myofibers to form new myotubes (Figure 2). Myotubes formed from satellite cells migrating out of myofibers were stained for myosin heavy chain (MF20) to establish the total myotube area and myotube diameter (Figure 2). Satellite cells from adult mice formed more and bigger myotubes as compared with satellite cells from old mice (Figure 2a–c). miR-21 overexpression in satellite cells from adult mice led to the formation of fewer and smaller myotubes. However, miR-21 overexpression had no effect on myotube formation from satellite cells from old mice compared with mock-transfected cells (Figure 2a–c). Conversely, miR-21 inhibition (AM21) led to an increase in myotube diameter and area from satellite cells from both adult and old mice, and an increased area of myotubes from satellite cells from adult mice as compared with controls (Figure 2a–c). These data suggest that miR-21 may play a biologically relevant in myogenic differentiation of satellite cells, one of the processes contributing to muscle regeneration.

### 3.3. miR-21 Negatively Regulates Myoblast Viability

**Figure 3:**
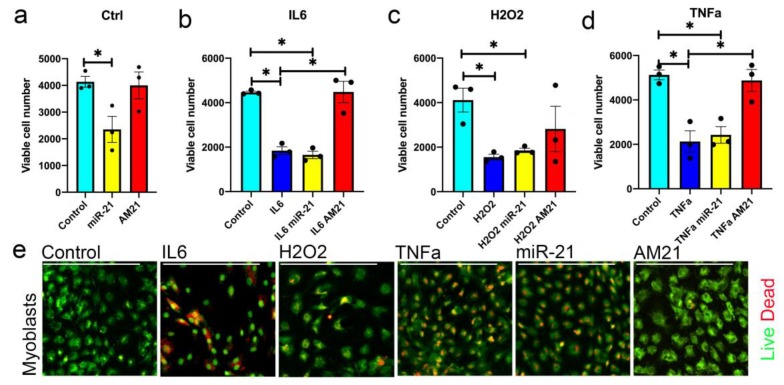
miR-21 regulates primary myoblast viability. (**a**–**d**) Myoblasts isolated from the adult mice were transfected with miR-21 mimic or inhibitor (AM21) and cultured in low serum media in the presence of 0.2 ng/mL interleukin 6 (IL6; **b**), 50 µM H_2_O_2_ (**c**) or 25 ng/mL TNFα (**d**) for 72 h, respectively. Cell metabolic activity indicative of cell viability was assessed via MTT assay. (**e**) Viable and dead myoblasts following transfections were visualised using live/dead staining (green—live cells, yellow/red—cells undergoing apoptosis/necrosis). Error bars show SEM, * *p* < 0.05 (compared to control), *n* = 3. Scale bar = 200 µm.

We next assessed whether miR-21 affects the potential of satellite cells to form new myotubes through regulation of their viability/proliferation. Primary myoblasts were transfected with miR-21 mimic or inhibitor, in the presence of IL6, TNFα or H_2_O_2_, respectively (Figure 3). An MTT assay was used to quantify metabolic activity as an indicator of the number of viable cells (Figure 3). Transfection of miR-21 in control conditions had a dramatic effect on the number of viable myoblasts (Figure 3a). Increased levels of miR-21 resulted in a decreased cell number and increased myoblast death as compared with control myoblasts (Figure 3a,e). However, miR-21 inhibition (AM21) did not have any effect on myoblast viability in control conditions (Figure 3a,e). We therefore analysed the effects of changes in miR-21 levels on myoblasts pretreated with H_2_O_2_, IL6 or TNFα. All treatments consistently resulted in decreased cell number and increased cell death of primary myoblasts as compared to control cells (Figure 3b–e). Overexpression of miR-21 in myoblasts treated with H_2_O_2_, IL6 or TNFα had no additional effect on the decrease in the number of viable myoblasts as assessed by MTT assay and live/dead staining (Figure 3b–e). This may be associated with already higher levels of miR-21 induced by H_2_O_2_, IL6 or TNFα treatment (Figure 3c–e). miR-21 inhibition in the presence of IL6 or TNFα promoted cell survival (Figure 3b,d,e). Inhibition of miR-21, however, had no effect on cell viability in the presence of H_2_O_2_ (Figure 3). 

Overall, these data suggest that miR-21 may primarily regulate the viability of mouse myoblasts in the context of a pro-inflammatory environment through mediating the effects of IL6 and TNFα.

### 3.4. miR-21 Regulates Myogenesis In Vitro

**Figure 4:**
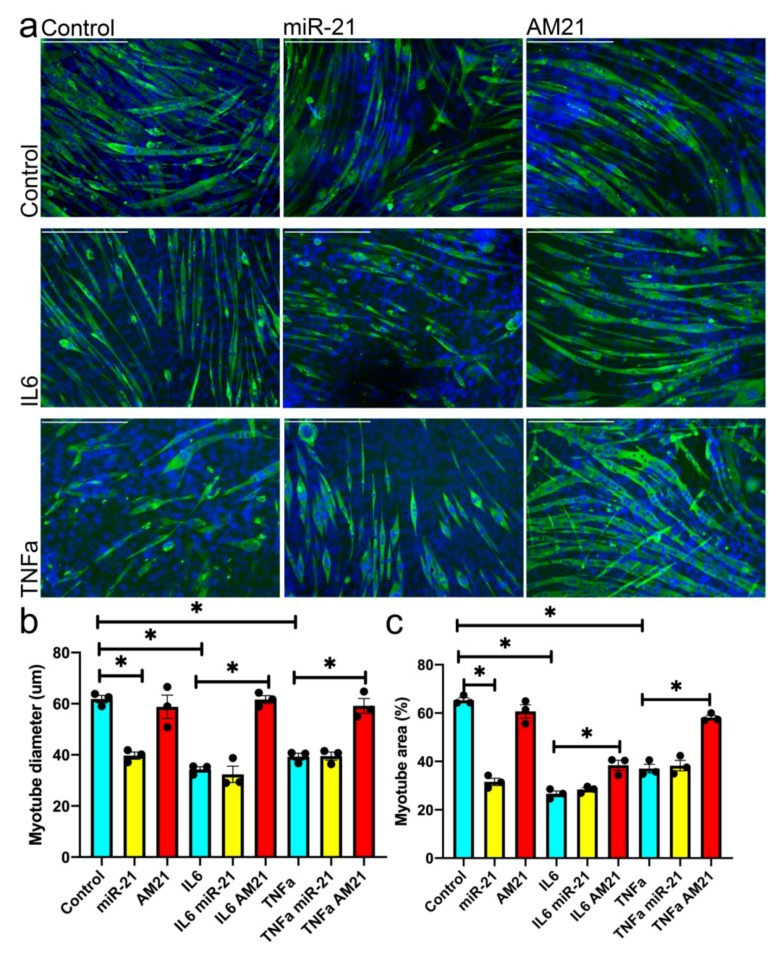
Inhibition of miR-21 positively regulates myogenesis in the presence of pro-inflammatory cytokines. (**a**) MF20 immunostaining showing myogenic differentiation regulation by miR-21 in the presence of 0.2 ng/mL interleukin 6 (IL6) or 25 ng/mL TNFα for 5 days, respectively. Expression of miR-21 was manipulated in undifferentiated myoblasts using miR-21 mimic or anti-miR-21 (AM21) in the presence of IL6, TNFα, respectively; control—mock-transfected cells. Myotubes were stained for myosin heavy chain: MF20—green; blue—DAPI. (**b**) Quantification of myotube diameter is shown. (**c**) Quantification of total myotube area is shown (%). Error bars show SEM, • indicates individual replicates, * *p* < 0.05 (compared to control), *n* = 3. Scale bar = 400 µm.

We next investigated whether the effects of miR-21 on satellite cell ability to form new myotubes is associated with miR-21 regulation of myogenic differentiation. miR-21 overexpression in mouse primary myoblasts resulted in the formation of fewer and smaller myotubes, whereas inhibition of miR-21 had no effect on myotube formation (Figure 4a–c). 

Since our data demonstrated the relevance of miR-21 in a pro-inflammatory environment, (Figure 3), we next investigated the potential of miR-21 to regulate myogenesis in vitro in the presence of TNFα or IL6. Mouse primary myoblasts were treated with TNFα or IL6, respectively, for the course of differentiation (5 days) and both treatments resulted in the formation of fewer and/or smaller myotubes (Figure 4). Overexpression of miR-21 in the presence of TNFα or IL6 had no additional effect on myotube formation as compared to TNFα and IL6-treated myoblasts, respectively (Figure 4a–c). However, inhibition of miR-21 (AM21) in the presence of either IL6 or TNFα, rescued the TNFα- and IL6-induced phenotype resulting in formation of more and bigger myotubes, as compared to TNFα or IL6-ttreated myoblasts, respectively (Figure 4a–c).

These data further support the hypothesis that miR-21 may play a role in regulating the efficiency of myogenic differentiation and therefore muscle regeneration in the context of a pro-inflammatory environment, which has been demonstrated previously during ageing [3].

### 3.5. miR-21 Regulates the Expression of IL6R and PTEN

**Figure 5:**
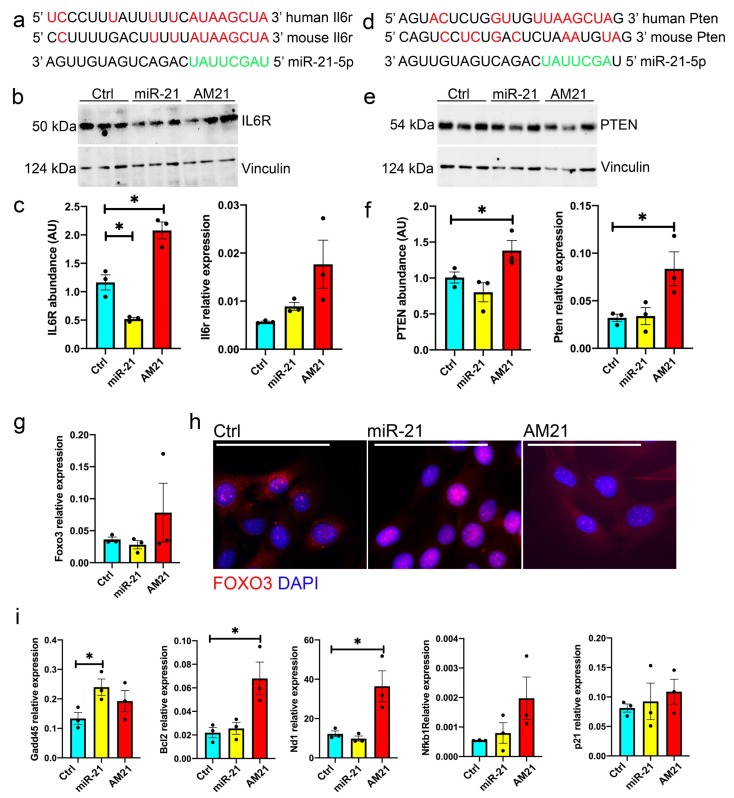
miR-21 regulates the expression of Il6r in mouse primary myoblasts. (**a**,**d**) Alignment of putative miR-21 target site in the 3′UTR of Il6r and Pten genes, respectively; human and mouse sequences are indicated; conserved miR-21 putative target site is indicated in green; complementary nucleotides are shown in red. (**b**,**c**) Primary mouse myoblasts were transfected with miR-21 mimic or antimiR (AM21). Endogenous IL6R protein but not mRNA expression is regulated by miR-21 in the mouse myoblasts, as shown by Western blot and qPCR, respectively. (**e**,**f**) Inhibition of miR-21 leads to upregulation of Pten mRNA and protein levels as demonstrated by Western blot and qPCR, respectively. (**g**–**i**) miR-21 does not significantly affect Foxo3 mRNA levels (**g**), however miR-21 upregulation results in translocation of FOXO3 into the nucleus (**h**) and upregulation of stress response gene: Gadd45 (**i**), whereas miR-21 inhibition (AM21) results in upregulation of antiapoptotic gene Bcl-2 and increase in mitochondrial marker Nd-1 as compared to mock-treated cells and miR-21 does not affect cell cycle regulator: p21 (**i**). Myoblasts treated with miR-21 or AM21 were stained for FOXO3 and DAPI; representative images shown; Expression relative to β-2 microglobulin, S18 and S29 shown. qPCR data show SEM; • indicates individual replicates, * *p* < 0.05 (compared to control—mock transfected cells); *n* = 3. Scale bar = 50 µm.

To understand miR-21 mechanism of action, we investigated putative miR-21 target genes. miR-21 has been previously shown to regulate the expression of IL6R and PTEN in the context of human cancer cells [42,43,44,45]. IL6R mediates some of the effects of IL6 in muscle and PTEN has been shown to be a downstream effector of TNFα [46,47]. We investigated the 3′UTR of Il6r and Pten for miR-21 binding sites: Il6r 3′UTR contains one putative miR-21 binding site conserved between human and mouse (Figure 5a), whereas Pten 3′UTR contains a putative binding site for miR-21, however this site did not seem to be fully conserved in mouse (Figure 5d). To validate Il6r and Pten as physiologically relevant miR-21 targets, we investigated the expression of Il6r and Pten mRNA and protein in primary mouse myoblasts following miR-21 overexpression or inhibition (AM21), respectively (Figure S1a and Figure 5b,c,e,f). The expression of Il6r mRNA was not significantly affected by miR-21 in mouse myoblasts, however miR-21 overexpression and inhibition led to downregulation and upregulation of IL6R protein, respectively, (Figure 5b,c). Overexpression of miR-21 did not result in changes of Pten mRNA or protein as compared to mock-transfected myoblasts, whereas miR-21 inhibition led to an upregulation of both Pten mRNA and protein levels. These results suggest that in murine myoblasts, Pten may be regulated by miR-21 in an indirect manner in mouse myoblasts, rather than through binding to Pten 3′UTR (Figure 2e,f).

miR-21:Pten interactions have been previously shown to regulate human cell viability via FoxO3 phosphorylation [43]. We found no effects of miR-21 on the levels of FoxO3 mRNA and protein but miR-21 suggested increased phosphorylation of FoxO3 in mouse primary myoblasts (Figure 5g and Figure S1c). We next investigated whether miR-21 upregulation results in translocation of FOXO3 into the nucleus. Myoblasts treated with miR-21 indicated nuclear localisation of FOXO3 suggesting activation of cell stress response/apoptosis pathway (Figure 5h). Consistently, an increase in miR-21 and concomitant nuclear FOXO3 localisation were associated with an increase in markers of cellular stress and apoptosis. The mRNA level of the DNA inducible damage protein Gadd45 was upregulated in myoblasts treated with miR-21, whereas the antiapoptotic gene Bcl-2 and mitochondrial marker Nd-1, were upregulated upon myoblast treatment with AM21, as compared to mock-transfected myoblasts (Figure 5i).

NF-kB has previously been shown to be upregulated in response to the inflammatory environment and contribute to muscle wasting [48]. Moreover, miR-21 is directly transcriptionally regulated by NF-kB [49]. Therefore, we investigated the potential regulation of NF-kB expression via miR-21. However, primary myoblasts treated with miR-21 mimic or AM21 did not show significant changes in Nfkb1 mRNA expression or p50 and p105 protein levels (Supplementary Figure S1d). We also did not observe any changes in AKT or phosphorylated AKT protein (Supplementary Figure S1b) or the expression of the cell cycle regulator p21 (Figure 5i) despite PTEN being an upstream regulator of these pathways.

## 4. Discussion

Progressive muscle loss during ageing and disease remains an unmet clinical need, as currently there are no effective interventions to regain muscle lost. The molecular mechanisms underlying muscle atrophy and the decline in muscle regeneration capabilities are still not fully understood. Changes in muscle and satellite cells at the mRNA and protein levels during ageing have been demonstrated in both humans and rodents [15,25,27]. Moreover, changes in the levels of miRs, post-transcriptional gene expression regulators, have also been demonstrated in muscle during ageing and disease [15,16,24,25,26,27]. microRNAs are predicted to regulate 2/3 of the human genome and are likely important regulators of ageing-related decline in muscle regeneration [15].

The role of miR-21 as an oncogene has been previously demonstrated in the context of cancer [50]. Interestingly, whilst the levels of circulating miR-21 has been shown to increase in older people, frail adults and patients with chronic disorders such as cardiovascular disease [31,32]. A recent publication by He et al. did not however observe significant changes in the levels of circulating miR-21 in sarcopenic older adults as compared to non-sarcopenic older adults [51]. These differences may be due to different populations studied and definitions used, further research is needed to validate the role of miR-21 as a potential biomarker of inflammation, frailty or sarcopenia.

In skeletal muscle, tumour-derived miR-21 has been shown to induce muscle atrophy [28]. Moreover, miR-21 has been shown to contribute to muscle atrophy following denervation through regulating YY1 protein and play an important role in muscle fibrosis during Duchenne muscular dystrophy [36,37].

In this study, we investigated whether miR-21 may contribute to the age-related decline in satellite cell function and potentially sarcopenia. Our data demonstrate that miR-21 was upregulated in muscle and satellite cells during ageing (Figure 1a,b). Moreover, miR-21 expression was elevated upon treatment of adult and old primary myoblast with H_2_O_2_, IL6 or TNFα (Figure 1c–e). This is consistent with previous finding indicating that ROS induce the expression of miR-21 through NF-kB activation [28].

Our data revealed differences in miR-21 expression following injury in the whole muscle between adult and old mice (Figure 1f,g). miR-21 basal levels were elevated in muscle from old mice (Figure 1f,g). Moreover, miR-21 levels were downregulated in regenerating muscle of adult mice at day 4, whilst miR-21 levels are upregulated in regenerating muscle of old mice (Figure 1f,g). This is consistent with previous data that demonstrate following injury an initial elevation of IL6 and TNFα in the muscle of adult and older rodents, while the levels of IL6 and TNFα decrease approximately at day 3 in regenerating muscle of adult mice but remain elevated in muscle of old mice [41]. Furthermore, in regenerating adult muscle, a switch from M1 to M2 macrophages and T cell recruitment occurs approximately at days 3–5 [52], a time point where we observed a downregulation of miR-21 expression in regenerating muscle from adult mice only (Figure 1f). This switch from pro- to anti-inflammatory is not as effective in regenerating muscle of old mice, hence it is possible that miR-21 is upregulated in regenerating muscle from old mice (Figure 1g) due to a chronic pro-inflammatory environment [7].

The differences in miR-21 between regenerating muscle from adult and old mice may also be associated with a disrupted feedback mechanism a chronic elevation of miR-21 with age that blunts the regeneration capacity. However, miR-21 expression could also be modulated by circulating cytokines and an altered myofibrillar environment during ageing, e.g., adipose tissue infiltration or changes in fibro-adipogenic progenitors.

Our data demonstrate that upregulation of miR-21 during muscle regeneration in vitro leads to formation of fewer and smaller myotubes from satellite cells migrating out of single fibres isolated from adult mice (Figure 2). Overexpression of miR-21 in satellite cells from old mice, which already have elevated levels of miR-21, had no effect on myotube size. Moreover, inhibition of miR-21 expression in satellite cells migrating out of single fibres isolated from old, but not adult mice, resulted in the formation of bigger myotubes, as compared to controls (Figure 2). Myogenic differentiation in our model is a result of satellite cell migration out of the fibres, their viability and myogenic potential. The phenotype observed is likely due to both changes in the number of viable myoblasts and myogenic potential and consistent with miR-21 overexpression leading to a decrease in viability and myogenic potential of primary myoblasts (Figure 3 and Figure 4).

Chronic exposure to low levels of IL-6 and TNFα has been previously shown to induce apoptosis in myoblasts [53]. Our data demonstrate that changes in miR-21 expression can attenuate alterations in the viable cell number in the presence of IL-6 and TNFα (Figure 3), although this could be a result of differences in myoblast proliferation or viability. However, our experimental design indicates the differences in myoblast viability contributes to the changes in viable cell number due to longer exposure of myoblasts to IL-6 and TNFα and viability being affected by miR-21 (Figure 3). Previous studies show contrasting data on miR-21 regulation of proliferation [38,54,55,56]. It remains to be established whether miR-21 regulates myoblast proliferation. Inhibition of miR-21 only had an effect on myoblast viability and myogenic potential when primary myoblasts were treated with IL6 or TNFα (Figure 3; Figure 4). We therefore propose that as miR-21 expression is upregulated upon stimulation of the cells with IL6 or TNFα, it may mediate the IL6- or TNFα-mediated myoblast death and decrease in myogenic potential phenotype. Therefore, a further increase in miR-21 levels produced by treatment of cells with miR-21 mimic, in cells pre-treated with IL-6 and TNFα, had no additional effects on myoblast viability and differentiation as miR-21 levels were already elevated in these cells (Figure 1c–e). However, inhibition of miR-21 function in myoblasts had a profound pro-survival and pro-myogenic phenotype in cells treated with IL6 or TNFα, further suggesting that miR-21 might be an important signaling molecule downstream of IL6 or TNFα.

We next investigated miR-21 target genes to elucidate the miR-21 function in primary myoblasts downstream of IL6 and TNFα. miR-21 has been previously shown to target IL6R and PTEN in human cells in the context of cancer [42,43,44,45]. TNFα has been shown to lead to upregulation of PTEN expression via NF-kB signalling [57]. Whilst TNFα treatment resulted in upregulation of miR-21 in primary myoblasts (Figure 1e), miR-21 overexpression did not result in changes in NF-kB or PTEN levels (Figure 5) suggesting that TNFα regulates the expression of these proteins independently of miR-21. However, our data show that inhibition of miR-21 leads to upregulation of PTEN (Figure 5). PTEN is a known negative regulator of the PI3K/AKT signaling cascade and as a result, promotes skeletal muscle atrophy and has been shown to regulate muscle protein degradation [58,59]. Despite upregulation of PTEN following inhibition of miR-21 levels in primary myoblasts (Figure 5), we did not observe changes in AKT or phosphorylated AKT (Figure S1) suggesting a potentially alternative mechanism of action. We also did not observe changes in p21 expression following miR-21 upregulation or inhibition, suggesting miR-21 does not regulate cell cycle, but rather cell viability (Figure 3 and Figure 5i). Independently of its regulation of AKT pathway, PTEN has been shown to regulate satellite cell quiescence and differentiation: conditional loss of Pten in myogenic progenitors can result in muscle hypertrophy, however at the cost of age-dependent exhaustion of satellite cells [60]. In line with this, PTEN has been shown to be necessary for satellite cell quiescence [61]. Therefore, the role of PTEN in muscle regeneration and wasting appears context-dependent, similar to the regulation of muscle regeneration in vitro by miR21 in pro-inflammatory conditions (Figure 2–4) [36]. It remains to be established whether miR-21 can regulate PTEN expression in vivo, in a pro-inflammatory environment.

Despite no changes observed in AKT and phosphorylated AKT levels following miR-21 overexpression or inhibition, miR-21 upregulation resulted in changes in translocalisation of FOXO3 to the nucleus (Figure 5g–i). This may be associated with IL6 signalling, as chronic IL6 overexpression can result in muscle atrophy by signalling through its receptor IL6R, activating FoxO3 [62]. FoxO3 has been described as the main initiator of the transcription of genes involved in protein degradation during muscle atrophy or atrogenes, e.g., MuRF-1 or Atrogin-1 [63]. Our data demonstrate that miR-21 is upregulated in myoblasts treated with IL6 and therefore may mediate IL6-induced FOXO3 activation in myoblasts. FOXO3 has been shown to regulate the cell stress response and apoptotic signaling [64]. Our data show an increase in Gadd45 levels following miR-21 upregulation concomitant with FOXO3 nuclear localisation (Figure 5h,i). Increased Gadd45 was previously shown to regulate muscle atrophy through activating proteolysis pathways and regulating the levels of the mitochondrial biogenesis regulator Pgc-1a [65,66]. Consistently, our data demonstrate that inhibition of miR-21 in myoblasts was associated with increased levels of anti-apoptotic factor Bcl-2 and increased levels of mitochondrial marker Nd-1, further suggesting the inhibition of miR-21 may lead to upregulation of pro-survival cellular pathways.

The role of IL6 in muscle wasting has been previously demonstrated [67]. Low levels of IL6 can promote activation of satellite cells and myotube regeneration, such as during exercise [67]. However, chronically elevated IL6 has been proposed to induce protein catabolism and muscle wasting during ageing and cachexia [67]. These distinct effects have been partially attributed to a crosstalk of the IL6/IL6 receptor and gp130 trans-signaling pathway that oppose to regenerative and anti-inflammatory of the classical IL6 receptor signaling pathway [67]. Upregulation of IL6R has been previously shown to have a pro-survival effect [68]. The activation of NF-kB in response to DNA damaging agents has been reported to upregulate IL6 and together with STAT3 increase the expression of miR-21 [69]. Interestingly, our data indicate that miR-21 regulates the expression of IL6R in primary myoblasts whose miR-21 binding site is conserved between humans and mice (Figure 5). It remains to be established whether IL6, miR-21 and IL6R form a feedback loop regulating muscle regeneration or atrophy. The effects of miR-21 on IL6 signalling could be mediated by modulating the expression IL6R and redirecting signaling pathways downstream of IL6 resulting in the activation of FoxO3 and pro-apoptotic pathways. Similarly, it remains to be established whether miR-21 mediates the effects of TNFα via PTEN regulation or alternative signaling molecules.

In conclusion, our study demonstrated that miR-21 played an important role in controlling myogenesis in vitro and potentially muscle regeneration. We proposed that the changes in miR-21 levels in satellite cells during ageing might act as a part of a regulatory mechanism resulting in a decline in satellite cell function. Whilst our data demonstrate the phenotypic effects of miR-21 in satellite cells and myoblasts from adult and old mice, it remains to be confirmed whether miR-21 acts via regulating IL6R and Foxo3 in myoblasts from old mice. It also remains to be established whether miR-21 can regulate myoblast proliferation, not only viability, as contrasting data have been previously published [55,56,70]. However, we did not observe changes in the expression of cell cycle regulator: p21 following miR-21 upregulation or inhibition (Figure 5i).

Interestingly, increased expression of miR-21 in skeletal muscle has been demonstrated not only in age related atrophy but also cachexia. Cachectic patients are also characterised by elevated levels of circulating cytokines, such as TNFα and IL-6 resulting in muscle protein catabolism and reduced muscle protein synthesis [2]. Our results would support previous findings [28,36,37] that identify miR-21 as playing an important role in regulating muscle wasting in inflammatory conditions, such as ageing and cachexia.

Our data, together with previous reports of miR-21 role as an inflamma-miR and oncogene, suggest that miR-21 inhibition may hold a therapeutic potential in the context of inflammation-related muscle wasting, such as during ageing or cancer cachexia. However, there are a number of limitations with the present study in relation to specifically studying miR-21 expression in satellite cells from adult and old mice. The expression of miR-21 is up regulated in satellite cell in pro-inflammatory conditions such as ageing and injury, yet it also modulates regulators of downstream signaling pathways indicating a regulatory feedback mechanism. In order to determine the specific role of elevated miR-21 in satellite cells that result in defective muscle regeneration, the regeneration capacity of old mice where we have specifically inhibited miR-21 in satellite cells from old animals would be required. It also remains to be determined whether the inhibition of miR-21 in satellite cells and/or muscle at the correct time point during lifespan, may override the effects of age-related changes such as chronically elevated levels of IL6 or TNFα, improving the regenerative potential of satellite cells in vivo.

## 5. Conclusions

In conclusion, miR-21 expression is upregulated in satellite cells during ageing and in response to pro-inflammatory cytokines: TNFα and IL-6. Our data show that miR-21 regulates the ability of satellite cells to form new myotubes in vitro, through controlling myoblast viability and differentiation. miR-21 is upregulated by pro-inflammatory cytokines: TNFα and IL6, and it likely regulates the effects of these cytokines through controlling the levels of IL6R and Pten. Inhibition of miR-21 in pro-inflammatory environment preserved myoblast viability and myogenic potential, whereas miR-21 upregulation results in nuclear localisation of FOXO3 and upregulation of stress response pathway, which is associated with reduced myoblast viability. Inhibition of miR-21 may represent a novel intervention for preserving muscle in the pro-inflammatory environment, such as during ageing or cachexia, however further in vivo studies are needed.

## Figures and Tables

**Table 1:** Sequences of primers used for qPCR.

Gene Name	Forward Primer Sequence	Reverse Primer Sequence
*β2-microglobulin*	GGAGAATGGGAAGCCGAACA	TCTCGATCCCAGTAGACGGT
*S29*	GGCAGTACGCGAAGGACATA	CAAGGTCGCTTAGTCCAACTTA
*S18*	CGGCTACCACATCCAAGGAAGG	CCCGCTCCCAAGATCCAACTAC
*Foxo-3*	AGTGGATGGTGCGCTGTGT	CTGTGCAGGGACAGGTTGT
*IL-6r*	CTTGGATAGCAGAGCCCAGG	CTCGTGGTTGGCAGAGTCTT
*Pten*	TTGGCGGTGTCATAATGTCT	GCAGAAAGACTTGAAGGCGTA
*Nfkb1*	ACACGAGGCTACAACTCTGC	GGTACCCCCAGAGACCTCAT
*P21*	GGCAGACCAGCATGACAGATTTC	CGGATTAGGGCTTCCTCTTGG
*Nd-1*	CCTATCACCCTTGCCATCAT	GAGGCTGTTGCTTGTGTGAC
*Gadd45*	CTGTGTGCTGGTGACGAACC	TCCATGTAGCGACTTTCCCG
*miR-21*	Cat. 218300	Universal Primer, part of cat. 218073
*Rnu-6*	Cat. MS00033740	Universal Primer, part of cat. 218073
*Snord-61*	Cat. MS00033705	Universal Primer, part of cat. 218073

**Table 2:** List of antibodies used in the study.

Reagent or Resource	Source	Identifier
Foxo3	Cell Signalling	2497
Phospho-Foxo3	Cell Signalling	9465
Pten	Cell Signaling Technology, Boston, USA	9188S
IL-6R	Abcam, Cambridge, UK	ab83054
Vinculin	Abcam, Cambridge, UK	ab18058
NF-kB	Abcam, Cambridge, UK	ab32360
MF20	Developmental Studies Hybridoma Bank, Iowa, USA	MF20 supernatant
IRDye 800CW Goat anti-Rabbit IgG	Li-Cor Biosciences, Cambridge, UK	926-32211
IRDye 800CW Goat anti-Mouse IgG	Li-Cor Biosciences, Cambridge, UK	926-68020
anti-mouse –Alexa488	ThermoFisher Scientific, Altrincham, UK	A28175

## Supplementary Materials

The following are available online at https://www.mdpi.com/2076-3921/9/4/345/s1, Figure S1: miR-21 does not affect AKT or NFkB signalling in mouse primary myoblasts. (**a**) miR-21 expression in primary myoblasts following treatment with miR-21 mimic or antagomiR (AM21). qPCR shows expression relative to Rnu-6; *n* = 3. Error bars show SEM. * *p* < 0.05. (**b**,**c**) miR-21 and has no effect no effect on AKT and phosphorylated AKT (P-AKT) levels or Nfkb1 expression as demonstrated by Western blot. (**d**) miR-21 upregulation may lead to increase in P-FoxO3 but not FoxO3 protein levels. • indicates individual replicates, Error bars show SEM; * *p* < 0.05.

## Appendix A

**Table A1:** Chemicals used in this study.

Reagent or Resource	Source	Identifier
DMEM	Sigma Aldrich, Dorset, UK	D5796
Foetal bovine serum	Sigma Aldrich, Dorset UK	F7524
Horse serum	ThermoFisher Scientific, Altrincham, UK	26050088
BSA	Sigma, Arklow, Ireland	A2153
Glycine	Sigma, Arklow, Ireland	G8898
Triton X	Sigma, Arklow, Ireland	×100 (lab-grade) or T8787 (for mol biology)
DAPI, 1 μg/mL	Sigma Aldrich, Dorset UK	D9542
Bradford assay	BioRad, Deeside, UK	
Trypsin	Sigma, Arklow, Ireland	T4049
2-propanol	Sigma, Arklow, Ireland	I9516
Chloroform:isoamyl alcohol 24:1	Sigma, Arklow, Ireland	C0549
Hydromount	National Diagnostics, Hull, UK	HS-106
Trizol	ThermoFisher Scientific, Paisley, UK	15596026
miR-21-5p mimic miRScript	Qiagen, Manchester, UK	219600
miR-21-5p inhibitor miRScript	Qiagen, Manchester, UK	219300
Lipofectamine 2000	Life Technologies, Paisley, UK	11668027
Mouse IL-6	Life Technologies, Paisley, UK	PMC0063
Mouse TNFα	Sigma, Gillingham, UK	T7539

**Table A2:** Software used in this study.

Reagent or Resource	Source	Identifier
ImageJ	Imagej.nih.gov	ImageJ
Prism8	Graphpad	Prism8
Photoshop	Adobe	Photoshop CC release 2017.1.6
AxioVision Software version 4.8	Zeiss; licence: University of Liverpool	AxioVision Software version 4.8

**Table A3:** Commercial kits used in this study.

Reagent or Resource	Source	Identifier
SuperscriptII	ThermoFisher Scientific, Loughborough, UK	18064014
miRScript RT II	Qiagen, Manchester, UK	218161
Qiagent Quanti-Tech	Qiagen, Manchester, UK	Part of 218073
miRScript SybrGreen	Qiagen, Manchester, UK	218073
