# Supplementary material for: Inflamma-miR-21 Negatively Regulates Myogenesis during Ageing
Source: Antioxidants (Basel). 2020 Apr 23;9(4):345. doi: 10.3390/antiox9040345 (PMC7222422; doi:10.3390/antiox9040345)
Supplement: Supplementary file 1 [file antioxidants-09-00345-s001.pdf]

Supplementary Material:

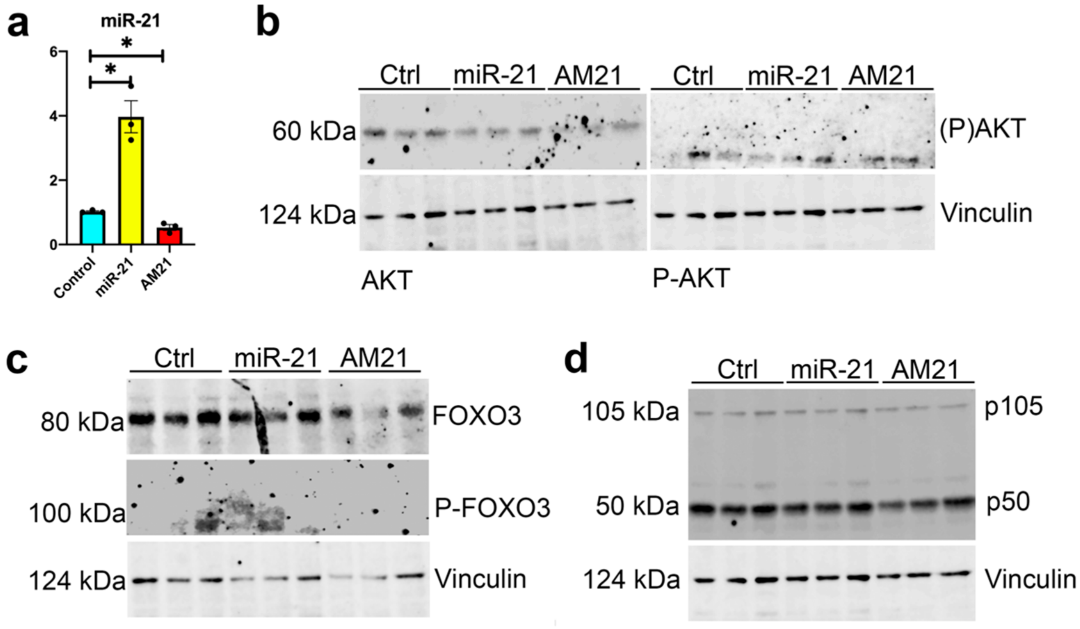

Figure S1. miR-21 does not affect AKT or NFkB signalling in mouse primary myoblasts. (**a**) miR-21 expression in primary myoblasts following treatment with miR-21 mimic or antagomiR (AM21). qPCR shows expression relative to Rnu-6; $n = 3$. Error bars show SEM. * $p < 0.05$. (**b,c**) miR-21 and has no effect no effect on AKT and phosphorylated AKT (P-AKT) levels or Nfkb1 expression as demonstrated by Western blot. (**d**) miR-21 upregulation may lead to increase in P-FoxO3 but not FoxO3 protein levels. • indicates individual replicates, Error bars show SEM; * $p < 0.05$.
